# Chronic kidney disease progression after cardiac surgery: a retrospective multicentre study

**DOI:** 10.1016/j.bjao.2025.100506

**Published:** 2025-12-09

**Authors:** Sune Bille, Rasmus B. Lindhardt, Lars P. Riber, Peter Juhl-Olsen, Hanne B. Ravn, Sebastian B. Rasmussen

**Affiliations:** 1 Department of Anaesthesiology and Intensive Care, Odense University Hospital, Odense, Denmark; 2 Department of Clinical Research, Health Faculty, University of Southern Denmark, Odense, Denmark; 3 Department of Cardiac, Thoracic and Vascular Surgery, Odense University Hospital, Odense, Denmark; 4 Department of Cardiothoracic and Vascular Surgery, Anaesthesia Section, Aarhus University Hospital, Aarhus, Denmark; 5 Department of Clinical Medicine, Aarhus University, Aarhus, Denmark

**Keywords:** acute kidney injury, cardiac surgery, chronic kidney disease, kidney failure, kidney function trajectory, long-term postoperative outcomes

## Abstract

**Background:**

Chronic kidney disease (CKD) is a well-established risk factor for adverse outcomes after cardiac surgery. However, the long-term trajectory of kidney function in this high-risk group remains poorly characterised. This study’s primary aim was to describe CKD progression and kidney failure in patients with kidney impairment before cardiac surgery. The secondary aim was to evaluate the impact of preexisting CKD in association with known risk factors (age, sex, and postoperative acute kidney injury) on CKD disease progression after cardiac surgery.

**Methods:**

This retrospective observational multicentre study included adult patients who underwent cardiac surgery at the Department of Cardiothoracic and Vascular Surgery, Odense University Hospital, between 2000 and 2022, and the Department of Cardiothoracic and Vascular Surgery, Aarhus University Hospital, between 2008 and 2024. Three outcomes were assessed, according to Kidney Disease: Improving Global Outcomes (KDIGO): rapid progression (confirmed estimated glomerular filtration rate [eGFR] decline ≥5 ml min^−1^ 1.73 m^2^ per year), CKD stage progression (confirmed decline in eGFR ≥25% with CKD stage advancement), and kidney failure (confirmed eGFR <15 ml min^−1^ 1.73 m^2^). Competing risk analysis accounted for mortality during median 7-yr follow-up.

**Results:**

Among 27 483 adult cardiac surgery patients, 3512 patients (12.8%) had preexisting CKD (KDIGO stages G3a–G5), based on preoperative eGFR levels. Five-year survival decreased with worsening baseline kidney function: 86.1% in stages G1–2, 70.6% in stage G3a, 61.4% in stage G3b, 45.7% in stage G4, and 51.9% in stage G5. Cumulative 5-yr incidence was 38.7% for rapid progression, 23.8% for CKD stage progression, and 5.5% for kidney failure. Events clustered early post discharge, with 43% of rapid progression, 26% of kidney failure, and 18% of CKD progression events occurring within the first year. Males aged ≤70 yr with stage G4 CKD who developed postoperative acute kidney injury faced highest risks across all outcomes.

**Conclusions:**

Cardiac surgery patients with preexisting CKD face substantial kidney disease progression, especially early after discharge. These findings highlight the need for research into structured follow-up programmes and kidney-preventive interventions.

Over the past decades, advances in cardiac surgery have significantly improved both short- and long-term mortality rates.^1^ However, cardiac surgery patients still face a high risk of postoperative complications, related to increasing age, burden of co-morbidities, and greater surgical complexity.^2^ Among these co-morbidities, chronic kidney disease (CKD) is a major risk factor and a growing global health concern, characterised by progressive decline in kidney function and an increased risk of cardiovascular-related hospitalisation and mortality, even in the earliest stages.^3^^,^^4^ Given the rising global prevalence of CKD and its increasing economic burden, early identification and intervention in high-risk populations becomes increasingly critical.^5^ In the context of cardiac surgery, preexisting CKD is a well-established risk factor for increased morbidity and mortality.^6^ In addition, CKD is closely associated with a higher risk of postoperative complications, including heart failure, arrythmia, respiratory failure, and infection.^7^, ^8^, ^9^ Furthermore, preexisting CKD significantly increases the risk of postoperative acute kidney injury (AKI)—a common and serious complication strongly associated with prolonged hospitalisation and both short- and long-term mortality.^10^, ^11^, ^12^ Importantly, given the high burden of AKI in this population, increasing attention has focused on prevention strategies, including standardised care bundles and novel pharmacological interventions targeting renal perfusion and metabolic reserve.^13^^,^^14^ Nevertheless, while the detrimental effects of preexisting CKD on short-term outcomes after cardiac surgery are well described, little is known about the long-term disease trajectories of kidney function in these patients.

The primary aim of the present study was to describe the incidence and timing of CKD progression and kidney failure in cardiac surgery patients with preexisting kidney impairment. Furthermore, the secondary aim was to evaluate the impact of CKD in association with known risk factors, such as age, sex, and postoperative AKI on CKD disease progression after cardiac surgery.

## Materials and methods

### Study population

This retrospective observational multicentre study included adult patients who underwent cardiac surgery at the Department of Cardiothoracic and Vascular Surgery, Odense University Hospital, between 1 January 2000 and 31 May 2022, and the Department of Cardiothoracic and Vascular Surgery, Aarhus University Hospital, between 19 May 2008 and 31 December 2024.

For patients with multiple cardiac operations during the study period, only the most recent procedure was included in the analysis.

The study was approved by the Regional Council of Southern Denmark (Journal no. 22/26952) and the Directorate of Region of Southern Denmark (Journal no. 22/39521). Because of the retrospective, registry-based study design, the requirement for written patient consent was waived.

### Data sources

Clinical data from the preoperative, intraoperative, and postoperative periods were extracted from the Western Denmark Heart Registry (WDHR), a database that has systematically collected information on all invasive cardiac procedures in Western Denmark since 1999.^15^ Inclusion of patients in the WDHR is mandatory for all cardiac procedures performed in the two included regions. Data quality is maintained through automated validation rules at the point of entry, complemented by systematic validation processes and random audits.

All Danish citizens are assigned a unique, lifelong 10-digit civil registration number, which facilitates linkage of patient data across individual health registries and laboratory systems. The WDHR is connected to the Danish Civil Registration System, which provides daily updates on mortality for all Danish citizens.^16^ This ensured that mortality was recorded for all patients up to the last follow-up on 31 May 2022 or 31 December 2024, depending on site.

Biochemical data for all patients, including in- and outpatient samples, were retrieved from regional laboratory systems at the Region of Southern Denmark and Central Denmark Region, respectively. These data spanned from 90 days before surgery until the last follow-up date using the civil registration number as the identifier.

### Baseline CKD status

We identified all available creatinine measurements from regional laboratory systems within 90 days before surgery. The baseline estimated glomerular filtration rate (eGFR) was calculated using the 2021 Chronic Kidney Disease Epidemiology Collaboration (CKD-EPI) equation^17^ using the lowest creatinine available in this period.

Patients were classified into CKD categories based on their baseline eGFR, following the 2024 Kidney Disease Improving Global Outcomes (KDIGO) definition^18^: stage G1–2 (eGFR ≥60 ml min^−1^ 1.73 m^2^), stage G3a (eGFR 45–59 ml min^−1^ 1.73 m^2^), stage G3b (eGFR 30–44 ml min^−1^ 1.73 m^2^), stage G4 (eGFR 15–29 ml min^−1^ 1.73 m^2^), and stage G5 (eGFR <15 ml min^−1^ 1.73 m^2^ or receiving dialysis treatment). Because of missing data, albuminuria measurements were not part of the CKD staging criteria.

### Kidney outcomes: rapid progression, CKD stage progression, kidney failure

In the analysis of CKD progression outcomes, patients classified as CKD stages G1–2 at baseline or those with preexisting kidney failure (stage G5) were excluded. Plasma creatinine samples obtained within the first seven days post surgery were excluded to minimise the influence of short-term AKI. All progression outcome assessments began 90 days post surgery to ensure evaluation of chronic rather than acute kidney function changes, though measurements from day 8 onward were available for slope calculations and longitudinal trend analysis.

Rapid progression was defined using a two-step approach. First, we identified potential rapid progression events as any eGFR measurement (m1) that was at least 5 ml min^−1^ 1.73 m^2^ lower than baseline eGFR, in accordance with KDIGO CKD guidelines. Second, we confirmed these events by requiring that the decline rate in the year leading up to and including m1 corresponded to an annual eGFR decline of at least 5 ml min^−1^ 1.73 m^2^ per year, calculated using linear regression analysis.

For the decline rate analysis, we required a minimum of three eGFR measurements from the year preceding m1, with these measurements spanning at least 90 days. The linear regression analysis included these preceding measurements plus m1 itself. Additionally, m1 required confirmation by a subsequent eGFR measurement obtained 30–180 days later. This confirmatory measurement had to either be ≤m1 or independently demonstrate the same decline criteria when analysed with its preceding measurements.

CKD stage progression was defined according to the KDIGO CKD guidelines as a decline of at least 25% in eGFR from baseline combined with advancement to a higher CKD category (i.e. from G3a to G3b, G3b to G4, or G4 to G5). This change required confirmation by a subsequent measurement taken at least 90 days later that maintained an eGFR within the range of the increased CKD stage and the minimum 25% eGFR reduction from baseline.

Kidney failure was established when a patient demonstrated at least two eGFR measurements below 15 ml min^−1^ 1.73 m^2^ (stage G5), with these measurements separated by a minimum of 90 days. For all three progression outcomes, the date of the outcome was defined as the date of the first confirmatory measurement that satisfied the respective criteria.

Kidney outcomes were assessed in patients with sufficient follow-up data, with incidence calculated relative to the entire population of 3144 patients with baseline CKD stages G3a–G4.

### Stratified risk analysis

This analysis further assessed several risk factors for kidney disease progression, based on our previous work identifying predictors of incident CKD development after cardiac surgery using explainable artificial intelligence methodology. From these findings, we selected age, sex, and postoperative AKI as key risk factors. AKI was defined according to KDIGO criteria as either a plasma creatinine increase of ≥26.5 μmol L^−1^ within 48 h or a ≥1.5-fold increase from baseline within seven days of surgery.^19^ Urine output was not available for AKI assessment. The risks of rapid progression, CKD stage progression, and kidney failure were analysed with stratification by baseline CKD stage.

### Statistical analysis

Continuous variables did not present Gaussian distributions and are presented as medians with 25th and 75th percentiles. Categorical variables are presented as counts and percentages. No imputation was performed for missing baseline covariate data. Patient follow-up began at the date of surgery and continued until death or the end of follow-up, whichever occurred first. During follow-up, we recorded the first occurrence of rapid progression, CKD stage progression, and kidney failure as separate outcomes.

Survival analysis was performed using the Kaplan–Meier estimator, with survival curves stratified by baseline CKD stage and compared using the log-rank test. *Post hoc* pairwise comparisons were performed between the reference group (CKD stage G1–2) and each of the other CKD stages. For CKD progression outcomes, we used the Aalen–Johansen estimator to calculate cumulative incidence while accounting for death as a competing risk, thereby appropriately addressing survivorship bias.

We constructed heatmaps to visualise the 1- and 3-yr risks of rapid progression, CKD stage progression, and kidney failure. These heatmaps were based on combinations of prespecified risk factors: age, sex, AKI occurrence, and baseline CKD stage.

To assess robustness of findings, sensitivity analyses were performed stratifying outcomes by AKI status and by surgery type (isolated coronary artery bypass grafting [CABG], isolated valve surgery, and combined procedures).

A two-sided *P*-value <0.05 was considered statistically significant. Statistical analyses were performed using R^20^ (version 4.2.0) and RStudio software^21^ (version 2024.4.2.764).

## Results

A total of 27 483 cardiac surgery patients were included for analysis (Supplementary Fig. S1). Of these, 23 971 patients were classified as CKD stages G1–2, while the remaining 3512 patients were distributed between CKD stages G3a to G5, with the majority in stage G3a. The median follow-up duration was 7.0 yr (25th–75th percentiles: 3.2–10.9 yr; range: 0.0–23.9 yr).

Baseline characteristics by CKD stages are presented in Table 1. Patients with CKD were generally older with a higher proportion of females compared with patients without kidney dysfunction, although the G5 group had a demographic profile comparable with patients with normal kidney function. Cardiovascular co-morbidities, including hypertension and diabetes, were more prevalent across all CKD stages. Surgically, CKD patients underwent fewer isolated CABG procedures and more often combination surgeries. The occurrence and severity of postoperative AKI increased progressively with worsening baseline kidney function, along with the need for kidney replacement therapy during intensive care unit (ICU) stay.

**Table 1:** Perioperative characteristics. Continuous data are expressed as median (25th–75th percentiles) and categorical variables are presented as counts (%) based on non-missing data. Age is expressed as mean (minimum–maximum). ACE, angiotensin-converting enzyme; AKI, acute kidney injury; CABG, coronary artery bypass grafting; CKD, chronic kidney disease; eGFR, estimated glomerular filtration rate; ICU, intensive care unit; KDIGO, Kidney Disease: Improving Global Outcomes; MI, myocardial infarction. Missing data: ∗971 patients, ^†^860 patients, ^‡^95 patients (all with preoperative dialysis dependency), ^¶^658 patients, ^§^692 patients, ^||^755 patients, ^#^404 patients, **∗∗**1 patient, ^††^271 patients, ^‡‡^6675 patients (note: dialysis during ICU stay was only registered from 2016 onward).

Variable	KDIGO CKD stage				
	G1–2 *n*=23971	G3a *n*=2249	G3b *n*=740	G4 *n*=155	G5 *n*=368
Sex (female)	5651 (24)	829 (37)	300 (41)	62 (40)	92 (25)
Age (yr)	66 (18–92)	73 (21–93)	73 (37–97)	70 (37–89)	61 (23–84)
Diabetes∗	3626 (16)	405 (19)	172 (24)	53 (36)	79 (22)
Hypertension^†^	13 155 (57)	1402 (65)	486 (69)	108 (73)	240 (68)
Baseline eGFR^‡^	90 (78–99)	54 (51–58)	40 (36–43)	25 (22–27)	15 (10–58)
ACE-inhibitor treatment^¶^	9336 (40)	1079 (50)	356 (50)	75 (51)	138 (41)
Ca-antagonist treatment^§^	6163 (26)	666 (31)	239 (34)	52 (36)	106 (32)
Previous MI^||^	4450 (19)	556 (26)	205 (29)	48 (32)	81 (23)
Previous cardiac surgery^#^	1450 (6)	167 (8)	63 (9)	13 (9)	38 (10)
Type of surgery∗∗					
- CABG	12 509 (52)	997 (44)	317 (43)	68 (44)	148 (40)
- Aortic valve	4968 (21)	457 (20)	159 (21)	30 (19)	72 (20)
- Mitral valve	1586 (7)	181 (8)	60 (8)	19 (12)	42 (11)
- Combinations	3262 (14)	454 (20)	140 (19)	21 (14)	69 (19)
- Other	1645 (7)	160 (7)	64 (9)	17 (11)	37 (10)
Overall AKI^††^	7017 (29)	1103 (50)	471 (64)	110 (73)	218 (80)
- Stage 1	4883 (69.6)	802 (72.7)	344 (73)	49 (44.5)	26 (11.9)
- Stage 2	1388 (19.8)	204 (18.5)	61 (13)	0 (0)	13 (6)
- Stage 3	746 (10.6)	97 (8.8)	66 (14)	61 (55.5)	179 (82.1)
Dialysis during ICU^‡‡^	186 (1)	28 (1)	20 (3)	7 (6)	48 (24)

### Mortality

Short-term survival decreased with advancing stages of preoperative CKD. Survival after 30 and 90 days was 97.5% and 96.8% in G1–2 patients, respectively, whereas survival in the G5 group was reduced to 86.7% and 84.8%, respectively (Supplementary Fig. S2, Supplementary Table S1).

**Fig 1:**
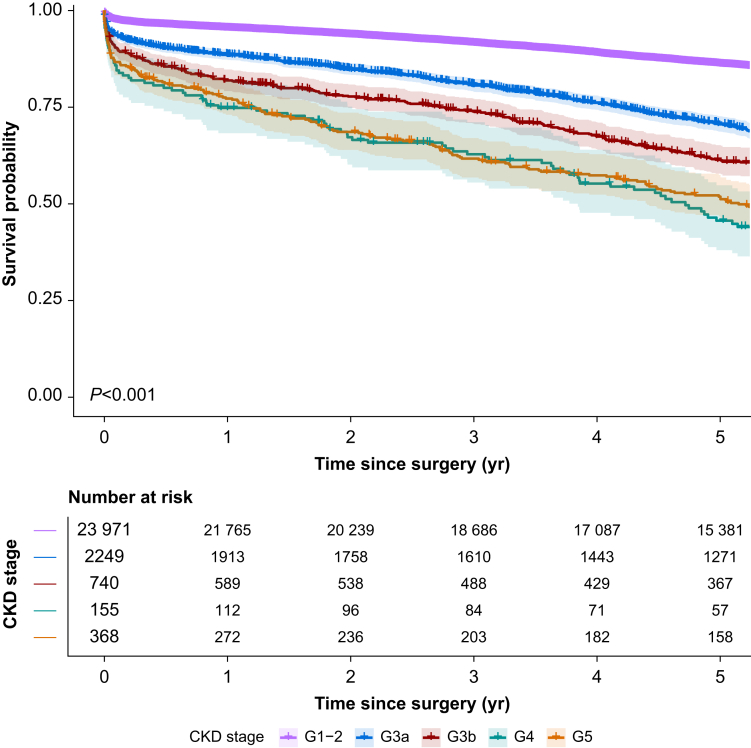
Survival curves. Kaplan–Meier survival curves showing 5-yr overall survival probability by preoperative chronic kidney disease (CKD), according to Kidney Disease: Improving Global Outcomes (KDIGO) classification. Shaded areas indicate 95% confidence intervals.

Long-term survival showed a similar pattern. Five-year survival was 86.1% in the G1–2 group but decreased progressively to 70.6%, 61.4%, 45.7%, and 51.9% in the G3a, G3b, G4, and G5 groups, respectively (Fig. 1, Supplementary Table S2). Pairwise comparisons of survival curves between the G1–2 reference group and each other CKD stage showed statistically significant differences (all *P*<0.001). Age-stratified analysis confirmed that this pattern persisted in both younger (≤70 yr) and older (>70 yr) patients (Supplementary Table S3).

### Rapid progression, CKD progression, and kidney failure

**Fig 2:**
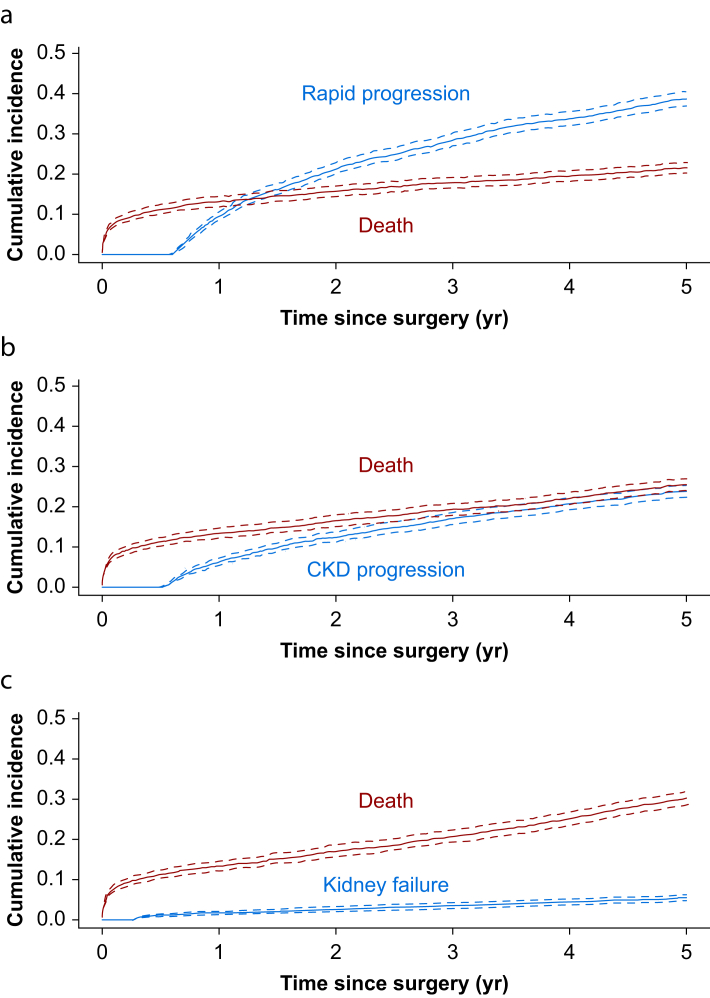
Cumulative incidence of kidney outcomes and death. Cumulative incidence curves showing competing risks of (a) rapid progression and death, (b) chronic kidney disease (CKD) progression and death, and (c) kidney failure and death over time after cardiac surgery. Grey lines represent the kidney-related events of interest, and red lines represent death as a competing event. Dashed lines indicate 95% confidence intervals.

Overall cumulative incidence of rapid progression, accounting for death as a competing risk, was 9.6% (303/3144; 95% confidence interval [CI] 8.6–10.7%) at 1 yr, 28.6% (898/3144; 95% CI 27.0–30.2%) at 3 yr, and 38.7% (1218/3144; 95% CI 37.0–40.5%) at 5 yr after surgery (Fig. 2a). For CKD progression, the cumulative incidence was 6.3% (198/3144; 95% CI 5.4–7.1%) at 1 yr, 16.9% (532/3144; 95% CI 15.6–18.3%) at 3 yr, and 23.8% (750/3144; 95% CI 22.3–25.4%) at 5 yr after surgery (Fig. 2b). The cumulative incidence of kidney failure increased from 1.6% (50/3144; 95% CI 1.1–2.0%) at 1 yr to 3.5% (110/3144; 95% CI 2.8–4.2%) at 3 yr, and 5.5% (172/3144; 95% CI 4.6–6.3%) at 5 yr after surgery (Fig. 2c). Temporal analysis of events over the entire follow-up period revealed that 43% of all rapid progression events, 18% of CKD progression events, and 26% of kidney failure events occurred within the first year after surgery (Supplementary Fig. S3).

### Risk factors for kidney disease progression outcomes

**Fig 3:**
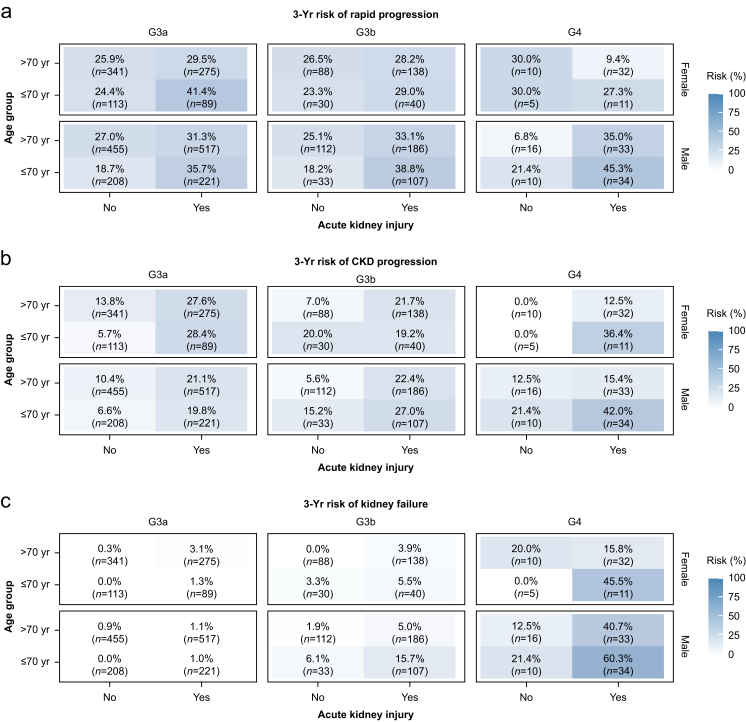
Three-year risk of kidney disease progression outcomes according to subgroups in competing risk analyses. Heatmaps showing 3-yr cumulative incidence of (a) rapid progression, (b) chronic kidney disease (CKD) progression, and (c) kidney failure from competing risk analyses stratified by baseline CKD stage (G3a, G3b, G4), age (≤70 or >70 yr), sex (female in upper panel or male in lower panel), and presence of acute kidney injury. Analysis includes 3144 patients with baseline CKD stages G3a–G4. The percentages in each cell represent the cumulative incidence for the respective outcome accounting for death as a competing event. The number in parentheses shows the number of patients in that specific subgroup. Colour intensity corresponds to the magnitude of risk, ranging from white (0%) to dark blue (100%).

Subgroup analysis of preselected variables revealed that the 3-yr risk of all outcomes varied substantially by CKD stage, age, sex, and postoperative AKI status (Fig. 3). For both rapid progression and CKD progression, the highest risks were observed in males aged ≤70 yr with stage G4 CKD who developed postoperative AKI (45.3% and 42.0%, respectively). AKI consistently increased progression risk across all CKD stages, with patients ≤70 yr generally showing higher progression rates than those older than 70 yr (Fig. 3a and b).

While the 3-yr risk of kidney failure was <5% in patients with CKD stages G3a and G3b, it increased in patients with CKD stage G4, particularly when combined with other risk factors. The highest risk (60.3%) was observed in males ≤70 yr with stage G4 CKD and postoperative AKI (Fig. 3c). Across all CKD stages, postoperative AKI was associated with increased risk of kidney failure, with this association being most pronounced in CKD stage G4.

Sensitivity analyses demonstrated that AKI significantly worsened survival across all CKD stages and substantially increased kidney progression risk. Among patients with baseline CKD stages G3a–G4, AKI increased the 3-yr risk of kidney failure nearly five-fold (1.2% *vs* 5.6%) and more than doubled CKD stage progression (10.1% *vs* 23.2%) (Supplementary Figs S4 and S5, Supplementary Tables S4 and S5). Additionally, stratification by surgery type demonstrated consistent patterns of kidney disease progression across isolated CABG procedures, isolated valve surgery, and combined procedures, with overlapping confidence intervals for all three outcomes (Supplementary Figs S6–S8, Supplementary Table S6).

## Discussion

This retrospective multicentre observational study revealed several critical insights into kidney disease trajectories in cardiac surgery patients with preexisting impaired kidney function. First, we found high rates of kidney disease progression, with 38.7% experiencing rapid progression, 23.8% CKD stage progression, and 5.5% developing kidney failure within 5 yr after surgery. Importantly, temporal analysis demonstrated that a substantial proportion of these events occurred within the first year after discharge, with 43% of rapid progression, 18% of CKD stage progression, and 26% of kidney failure events clustering in this early period. Second, risk stratification revealed that males aged ≤70 yr with stage G4 CKD who developed postoperative AKI faced the highest risk for all three outcomes. Finally, we found significantly reduced short- and long-term survival in patients with preoperative CKD, with an inverse relationship between survival and CKD stage severity observed both overall and in the age-stratified analysis. Consequently, despite the younger mean age in the G5 group, the excess mortality in advanced CKD represents a true effect of kidney disease rather than age differences.

Given that cardiac surgery patients represent a high-risk population with clinically significant cardiovascular disease, we compared our stage G3 CKD patients to a recent population-based study of incident CKD stage G3 patients. Despite similar baseline kidney function, several striking differences emerged: while mortality (21.0% *vs* 18.1%) and progression to a more advanced CKD stage (16.8% *vs* 14.3%) were similar at 3 yr, cardiac surgery patients showed substantially higher rates of rapid progression (28.7% *vs* 14.6%) and kidney failure (2.0% *vs* 0.3%).^3^ The present competing risk analysis identified postoperative AKI as the key driver of these differences. Among G3 patients without AKI, the 3-yr risk of rapid progression was comparable with that observed in the population-based study, whereas those who developed postoperative AKI demonstrated markedly elevated risks across all outcomes (Fig. 3).

This mechanistic relationship reflects the dual vulnerability of patients with preexisting kidney impairment, who face both a higher susceptibility to postoperative AKI and accelerated progression when AKI occurs.^22^ Additionally, the lower progression rates in patients >70 yr likely reflect competing mortality, where death events preclude observing kidney disease progression. Development of AKI can cause irreversible nephron loss and shorten kidney lifespan; cardiac surgery increases AKI risk through cardiopulmonary bypass-related haemodynamic alterations, inflammation, oxidative stress, and ischaemia–reperfusion injury.^23^ As an example, during cardiopulmonary bypass, renal oxygen delivery decreases by ∼20% as a result of reduced renal blood flow and haemodilution, creating an oxygen supply-demand mismatch that particularly affects the vulnerable renal medulla.^24^ This perioperative kidney injury appears to have immediate consequences, as temporal analysis revealed a distinct pattern of early progression among patients who developed kidney-deteriorating outcomes. The clustering of events within the first year suggests that perioperative insults to patients with already impaired kidney function may initiate rapid progressive dysfunction, accelerating the natural history of CKD in this high-risk cardiovascular population. This bidirectional relationship between AKI and CKD is consistent with findings from a recent systematic review and meta-analysis in cardiac surgery patients showing that postoperative AKI increases the odds of developing new-onset CKD by more than five-fold,^25^ establishing AKI as both a consequence of kidney vulnerability and a catalyst for further deterioration. In our cohort, sensitivity analyses stratified by AKI status directly quantified this association in patients with preexisting CKD, confirming AKI as an independent and potent driver of accelerated kidney disease progression. The high rates of progression, particularly among patients who developed postoperative AKI, underscore the urgent need for effective prevention strategies. Although several potentially modifiable risk factors have been identified, prevention remains challenging given the multifactorial aetiology of AKI.^23^ Nevertheless, recent evidence indicates that early risk stratification using urinary stress biomarkers, combined with targeted implementation of the KDIGO preventive care bundle, may reduce AKI incidence in high-risk cardiac surgery patients.^26^^,^^27^

The clinical implications of our findings are considerable. Over the last decades, several pharmacological options have emerged in the treatment of CKD, including the use of renin–angiotensin–aldosterone system (RAAS) inhibitors, which have been proved to delay CKD progression.^28^ More recently, treatment with sodium-glucose cotransporter 2 (SGLT2) inhibitors and glucagon-like peptide 1 (GLP-1) agonists—and most recent with SGLT2 inhibitors in combination with non-steroid mineralocorticoid antagonists—has demonstrated beneficial effects in delaying CKD progression, and reducing cardiovascular events and mortality.^29^, ^30^, ^31^, ^32^ However, these pharmacological treatments remain underutilised despite their proven benefits. Recently, a study on US adults with hypertension and albuminuria reported that even well-established therapies such as RAAS inhibitors are prescribed to only half of eligible diabetic patients and less than one-third of non-diabetic patients.^33^ Similarly, in our patient population, angiotensin-converting enzyme (ACE) inhibitors were prescribed to only ∼50% of patients with preoperative CKD. Given the rates of kidney disease progression, particularly within the first few years after discharge, these findings highlight the urgent need for structured follow-up programmes and early initiation of kidney-protective therapies in post-cardiac surgery patients with preexisting kidney disease.

Several methodological strengths support the validity of our findings. Use of competing risk analysis provides more accurate estimates of CKD progression risk by appropriately accounting for the high mortality rates in this population.^34^ Additionally, our conservative approach of using the lowest creatinine measurement within 90 days before surgery provided robust baseline kidney function estimates, which was especially important given that many patients were transferred from regional hospitals for urgent procedures (e.g. non-ST-elevation myocardial infarction) and may have developed kidney impairment before transfer. The long median follow-up period of 7 yr and comprehensive capture of outcomes through mandatory registry participation further strengthen our conclusions.

However, several limitations warrant consideration when interpreting these results. The retrospective design limits causal inferences and may introduce selection bias, including differential selection of healthier patients with advanced CKD for surgical *vs* less invasive endovascular alternatives, and availability of biochemistry data during follow-up. The G5 cohort exemplifies this selection bias, demonstrating a paradoxical demographic and outcome profile with younger age and better survival than the G4 group despite more advanced kidney disease. This likely reflects preferential selection of younger, healthier patients with end-stage renal disease for cardiac surgery, while older or frailer G5 patients were offered alternative management strategies. Additionally, the number of patients was limited in some of the subgroups of the competing risk analysis, which may affect interpretation and generalisability of risk stratification findings. The requirement for three or more creatinine measurements over ≥90 days to define rapid progression may have introduced selection bias toward patients with closer clinical follow-up. However, this criterion was necessary to distinguish true progression from acute fluctuations in kidney function. The absence of albuminuria data represents a significant limitation in our CKD classification, as current KDIGO guidelines emphasise the importance of both eGFR and urine albumin for CKD classification. Similarly, the absence of urine output also limits AKI assessment and classification. Furthermore, our study’s two-centre design solely in Danish cardiac surgery patients may limit generalisability to other healthcare settings and populations with different demographic or clinical characteristics.

In conclusion, cardiac surgery patients with preexisting kidney impairment face substantial risks of further decline in kidney function and increased mortality, with the highest risk within the first years after surgery. These results emphasise the need for further research to determine whether systematic kidney function monitoring and early implementation of kidney-protective medication in patients with preoperative CKD can minimise further decline in kidney function and improve outcomes.

## Authors’ contributions

Study design: all authors

Writing up of the first draft of the paper: SB

Analysis and interpretation of data: all authors

Final approval of the version to be published: all authors

Agreement to be accountable for all aspects of the work: all authors

Critical revision for important intellectual content: RBL, LPR, PJ, HBR, SBR

Acquisition of data: RBL, HRB, SBR

## Funding

This work was supported by a grant from The Lundbeck Foundation (R484-2024-1704).

## Declarations of interest

The authors declare that they have no conflicts of interest.
