# Supplementary material for: Chronic kidney disease progression after cardiac surgery: a retrospective multicentre study
Source: BJA Open. 2025 Dec 9;16:100506. doi: 10.1016/j.bjao.2025.100506 (PMC12753267; doi:10.1016/j.bjao.2025.100506)
Supplement: Multimedia component 1 [file mmc1.pdf]

Supplementary Materials

Chronic Kidney Disease Progression After Cardiac Surgery: A Retrospective Multicenter Study

Sune Bille*, Rasmus B. Lindhardt, Lars P. Riber, Peter Juhl-Olsen, Hanne B. Ravn, Sebastian B. Rasmussen*

*Corresponding authors: Sebastian B. Rasmussen (email: sebastian.buhl.rasmussen@rsyd.dk) and Sune Bille (email: sune.hansen3@rsyd.dk.)

This PDF file includes:

Figures S1 to S8
Tables S1 to S6

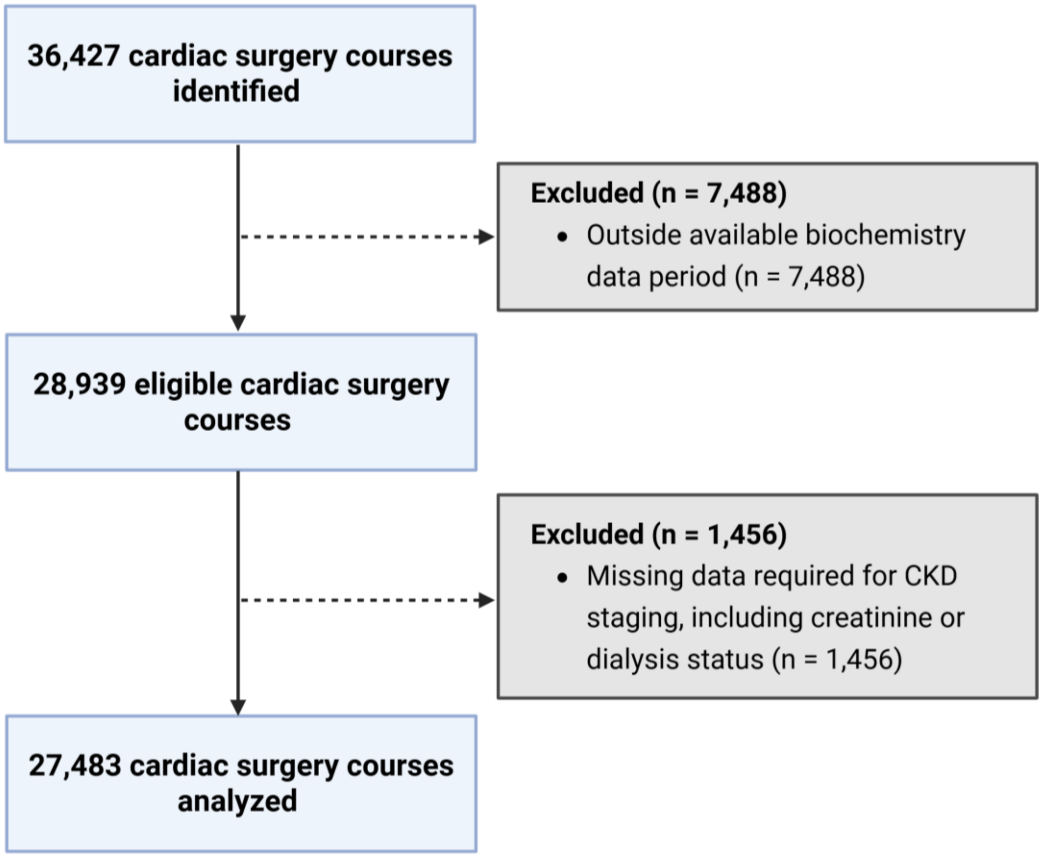

Figure S1. Study flow diagram. Flow chart showing patient selection for the study cohort. From 36,427 cardiac surgery courses identified in the Western Danish Heart Registry, 7,488 were excluded due to surgical procedures performed outside the available biochemistry data period (Aarhus University Hospital before May 19, 2008). Of the 28,939 eligible courses, 1,456 were excluded due to missing baseline creatinine measurements or information on chronic dialysis dependency required for baseline CKD staging, resulting in a final analysis population of 27,483 cardiac surgery courses. Abbreviations: CKD, chronic kidney disease (figure created in BioRender.com).

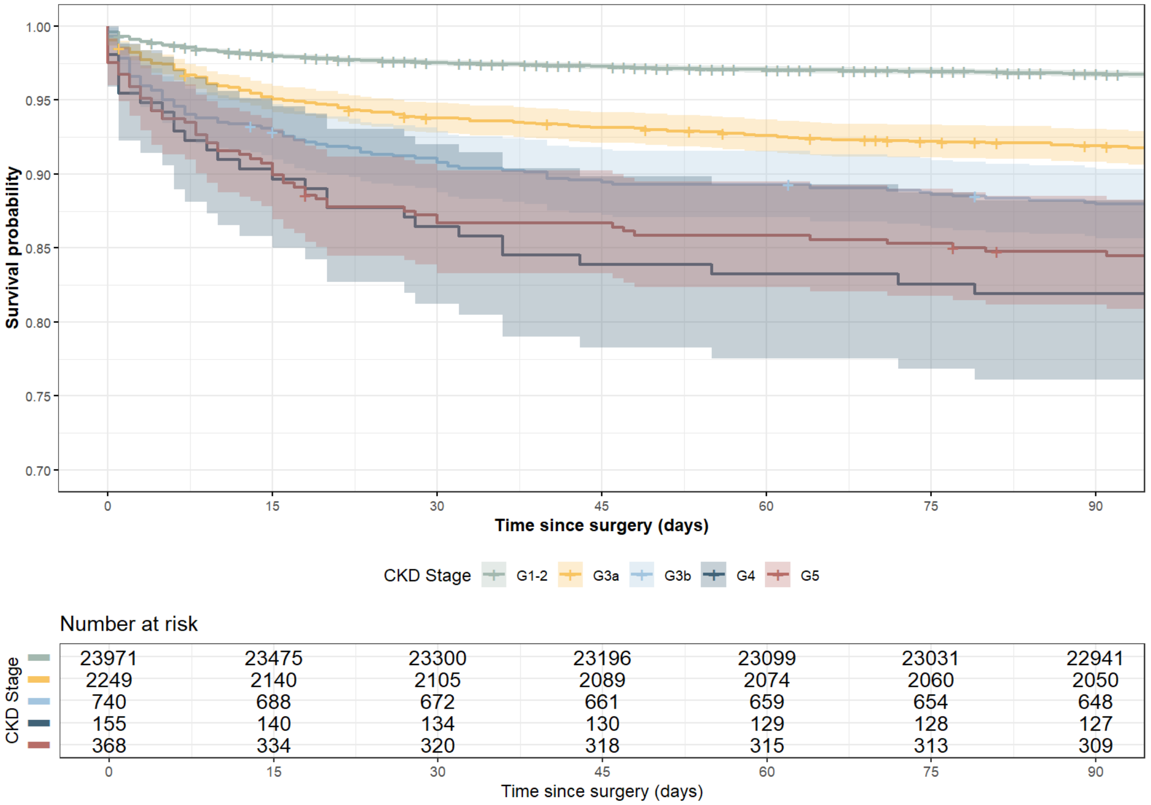

Figure S2. Short-term survival curves. Kaplan-Meier survival curves showing 90-day overall survival probability by preoperative chronic kidney disease (CKD) stage. Shaded areas indicate 95% confidence intervals.

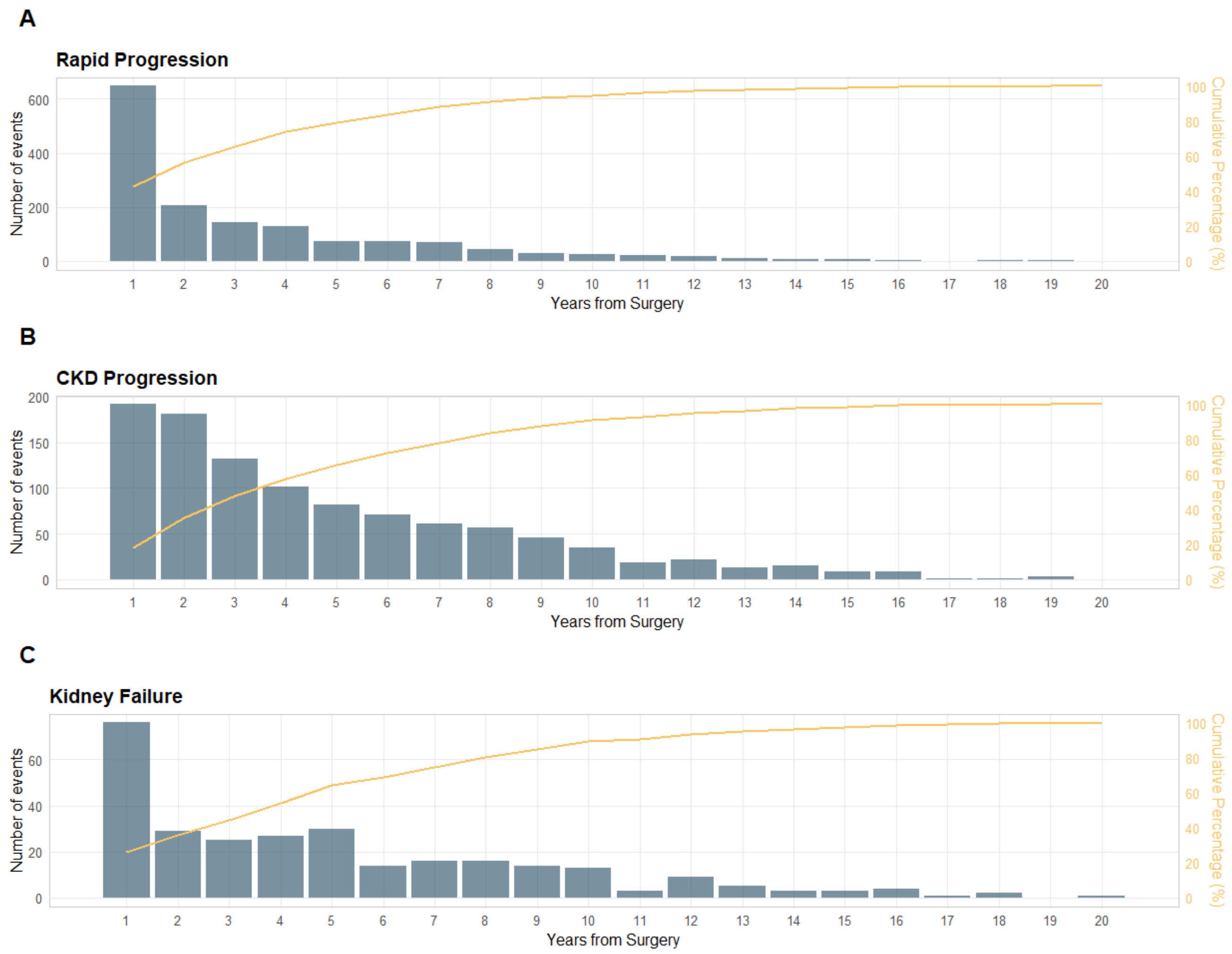

Figure S3. Temporal distribution of kidney outcomes after cardiac surgery. Bar charts showing the distribution of (**A**) rapid progression, (**B**) CKD progression, and (**C**) kidney failure events by year after surgery among patients who developed these outcomes. Blue bars represent the number of events occurring in each year, and orange lines show the cumulative percentage of total events over time. These analyses include only patients who developed the respective outcomes during follow-up and are not adjusted for competing risks.

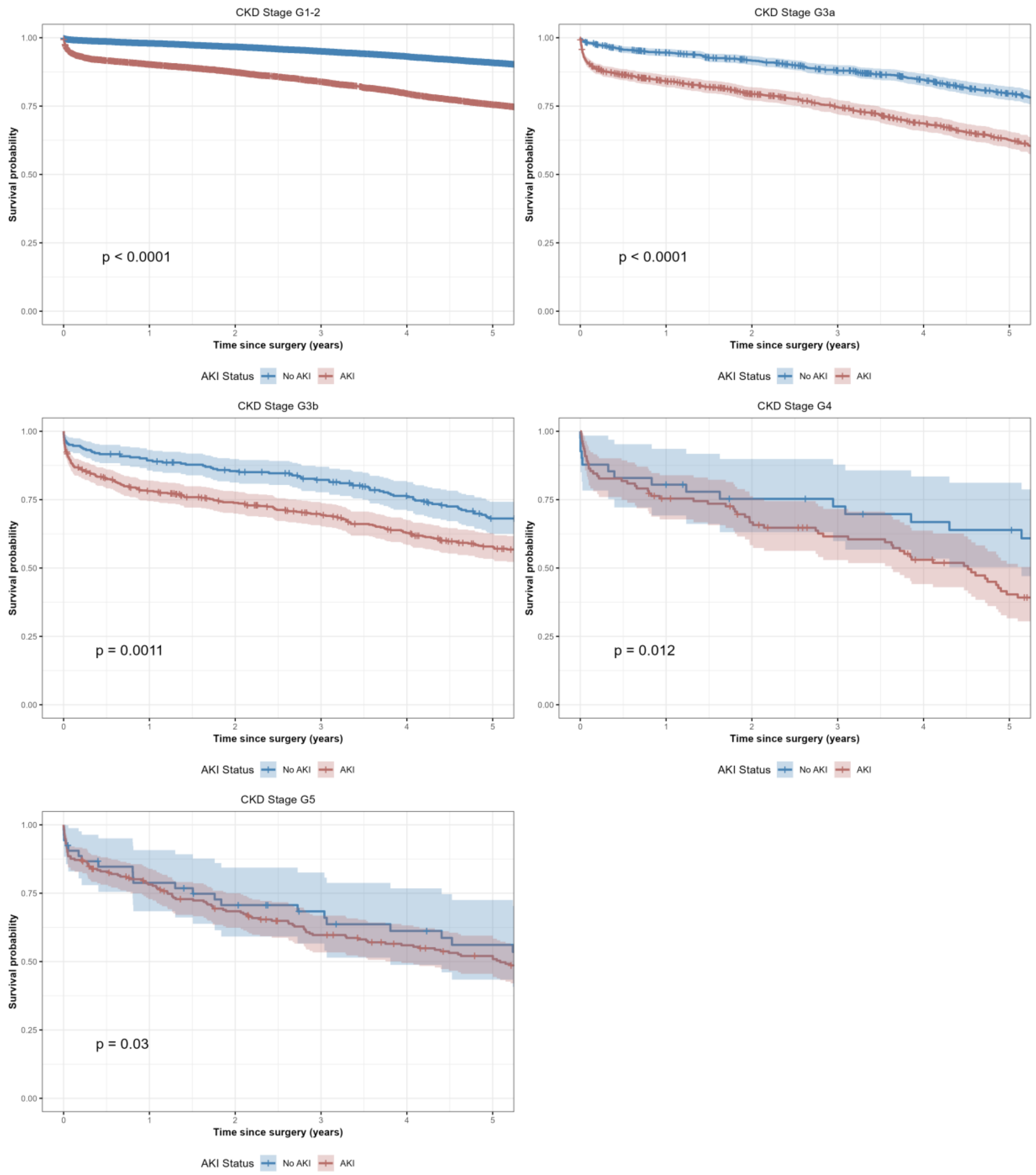

Figure S4. Impact of postoperative acute kidney injury on survival stratified by baseline CKD stage. Kaplan-Meier survival curves comparing patients with and without postoperative AKI across five baseline CKD stages: G1–2 (eGFR ≥60 mL/min/1.73 m²), G3a (eGFR 45–59), G3b (eGFR 30–44), G4 (eGFR 15–29), and G5 (eGFR <15 or dialysis). Blue lines represent patients without AKI, red lines represent patients with AKI. Shaded areas indicate 95% confidence intervals. P-values from log-rank tests are shown for each CKD stage. AKI was defined according to KDIGO criteria as either a plasma creatinine increase of ≥26.5 μmol/L within 48 hours or a ≥1.5-fold increase from baseline within seven days of surgery. Analysis includes 27,214 patients with available AKI data out of 27,483 total cardiac surgery patients. Abbreviations: AKI, acute kidney injury; CKD, chronic kidney disease; eGFR, estimated glomerular filtration rate.

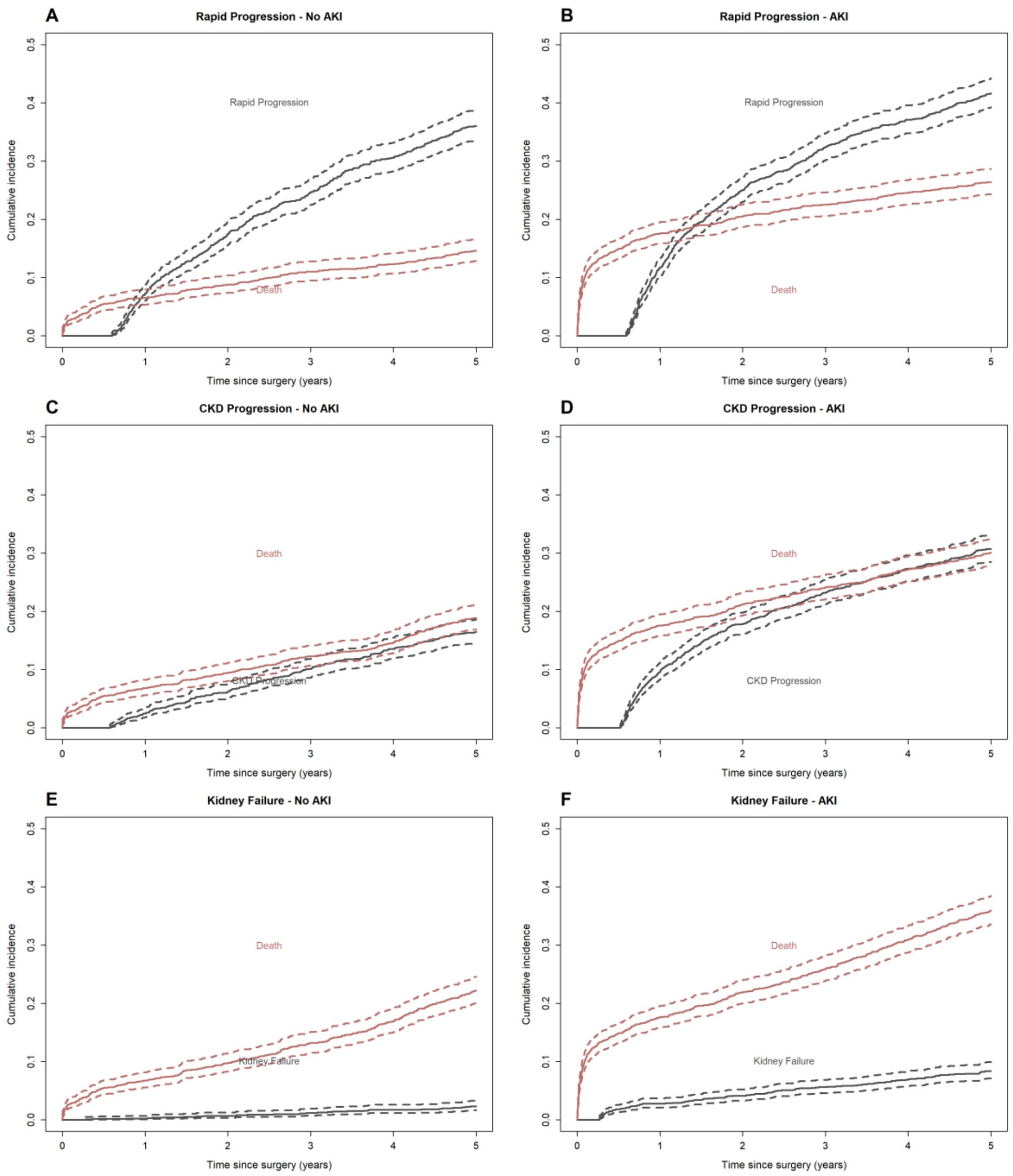

Figure S5. Competing risk analysis of kidney disease progression outcomes stratified by postoperative acute kidney injury status. Cumulative incidence curves showing the competing risks of kidney outcomes (gray/black lines) and death (red lines) over 5 years after cardiac surgery, stratified by presence or absence of postoperative acute kidney injury (AKI). Panels show: (**A**) rapid progression without AKI, (**B**) rapid progression with AKI, (**C**) chronic kidney disease (CKD) stage progression without AKI, (**D**) CKD stage progression with AKI, (**E**) kidney failure without AKI, and (**F**) kidney failure with AKI. Dashed lines indicate 95% confidence intervals. Curves were calculated using the Aalen-Johansen estimator to account for death as a competing event. Analysis restricted to 3,144 patients with baseline CKD stages G3a–G4 who had available AKI data and sufficient follow-up creatinine measurements to assess progression outcomes.

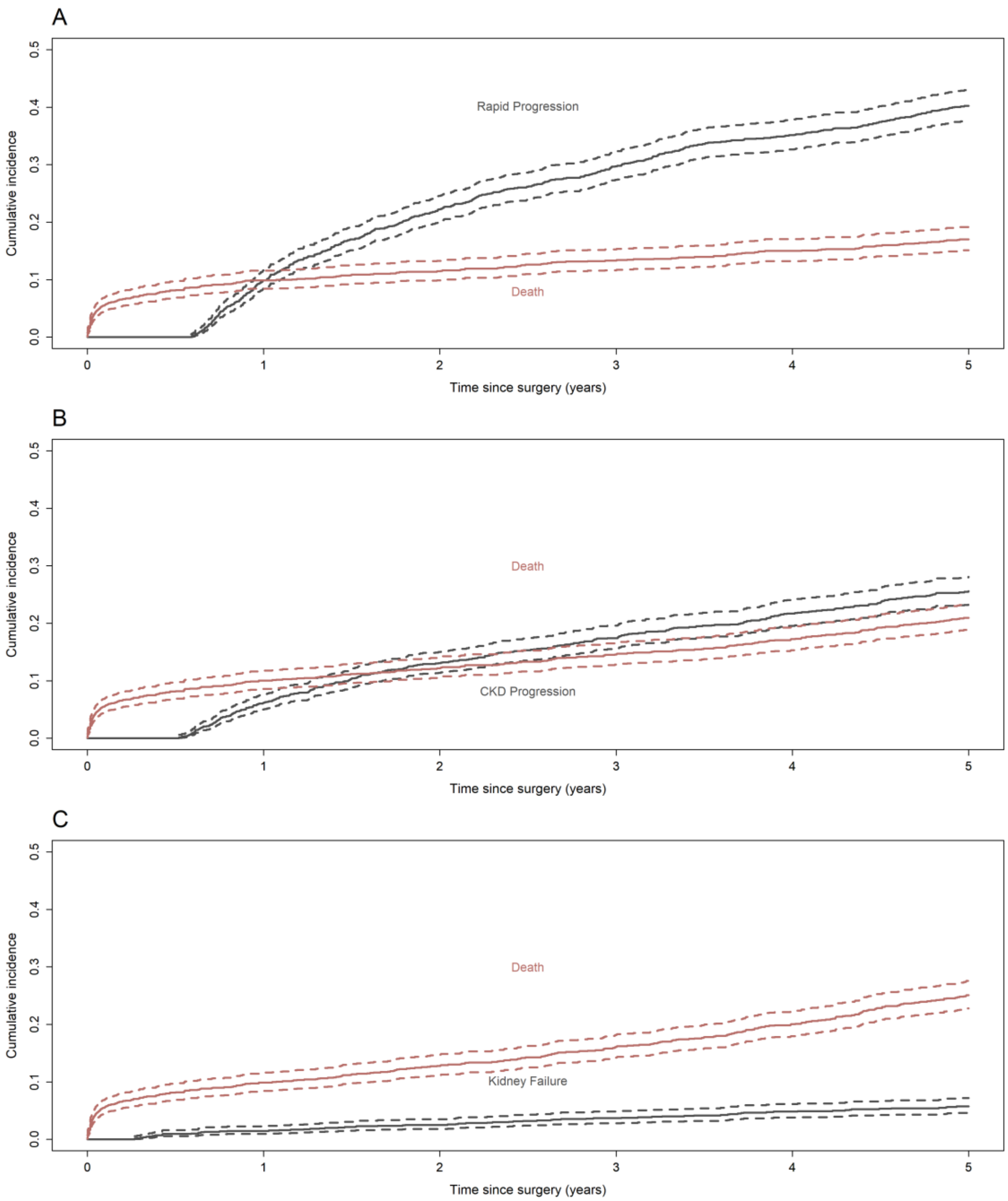

Figure S6. Competing risk analysis of kidney disease progression outcomes in patients undergoing isolated coronary artery bypass grafting (CABG). Cumulative incidence curves showing the competing risks of kidney outcomes (gray/black lines) and death (red lines) over 5 years after cardiac surgery in patients who underwent isolated CABG procedures. Panels show: (**A**) rapid progression, (**B**) chronic kidney disease (CKD) stage progression, and (**C**) kidney failure. Dashed lines indicate 95% confidence intervals. Curves were calculated using the Aalen-Johansen estimator to account for death as a competing event. Analysis restricted to 1,382 patients with baseline CKD stages G3a–G4 who underwent isolated CABG and had sufficient follow-up creatinine measurements to assess progression outcomes.

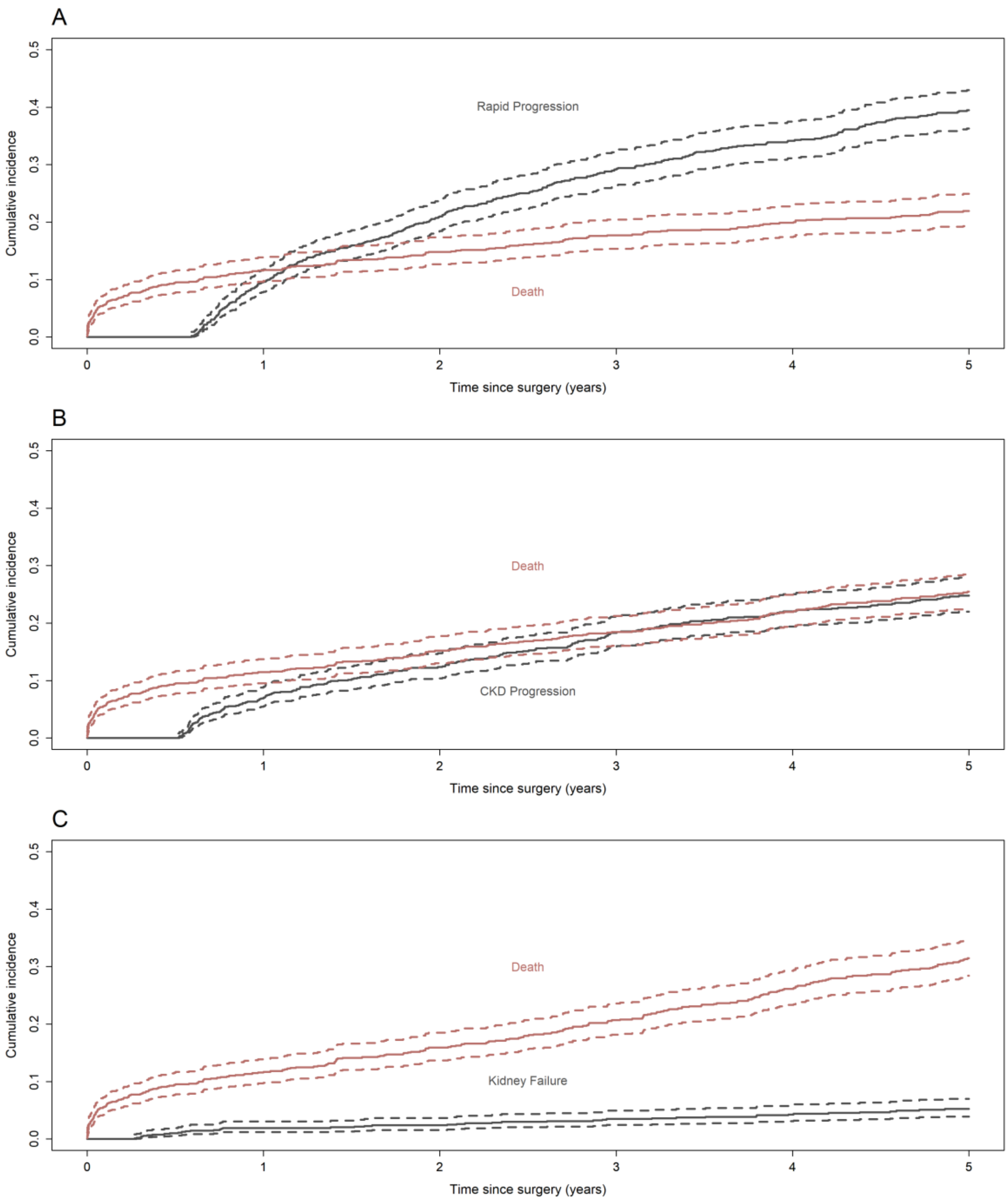

Figure S7. Competing risk analysis of kidney disease progression outcomes in patients undergoing valve surgery. Cumulative incidence curves showing the competing risks of kidney outcomes (gray/black lines) and death (red lines) over 5 years after cardiac surgery in patients who underwent aortic or mitral valve replacement/repair. Panels show: (**A**) rapid progression, (**B**) chronic kidney disease (CKD) stage progression, and (**C**) kidney failure. Dashed lines indicate 95% confidence intervals. Curves were calculated using the Aalen-Johansen estimator to account for death as a competing event. Analysis restricted to 906 patients with baseline CKD stages G3a–G4 who underwent valve surgery (646 aortic, 260 mitral) and had sufficient follow-up creatinine measurements to assess progression outcomes.

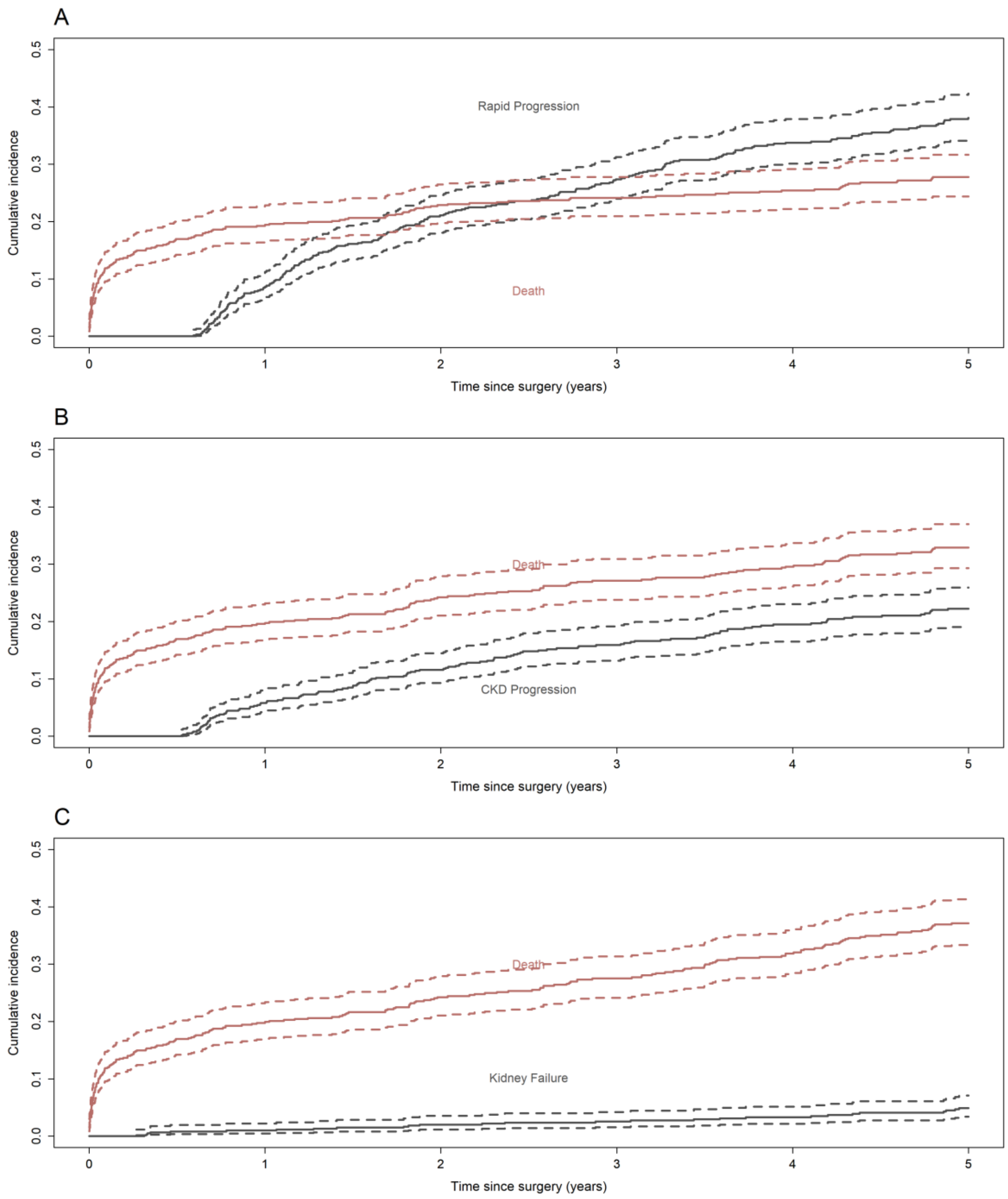

Figure S8. Competing risk analysis of kidney disease progression outcomes in patients undergoing combined cardiac procedures. Cumulative incidence curves showing the competing risks of kidney outcomes (gray/black lines) and death (red lines) over 5 years after cardiac surgery in patients who underwent combined procedures (typically CABG plus valve surgery). Panels show: (**A**) rapid progression, (**B**) chronic kidney disease (CKD) stage progression, and (**C**) kidney failure. Dashed lines indicate 95% confidence intervals. Curves were calculated using the Aalen-Johansen estimator to account for death as a competing event. Analysis restricted to 615 patients with baseline CKD stages G3a–G4 who underwent combined procedures and had sufficient follow-up creatinine measurements to assess progression outcomes.

CKD stage	30 days	90 days
G1-2	97.5% (97.3–97.7)	96.8% (96.6–97.0)
G3a	93.8% (92.8–94.8)	91.9% (90.8–93.1)
G3b	90.8% (88.7–92.9)	88.0% (85.6–90.3)
G4	86.5% (81.2–92.0)	81.9% (76.1–88.2)
G5	86.7% (83.3–90.2)	84.8% (81.2–88.5)

Table S1. Kaplan-Meier estimates of 30-day and 90-day survival (% with 95% confidence intervals) stratified by preoperative chronic kidney disease (CKD) stage.

CKD stage	Year 1	Year 3	Year 5
G1-2	95.4% (95.1–95.6)	91.5% (91.1–91.8)	86.1% (85.6–86.6)
G3a	88.6% (87.2–89.9)	80.7% (79.1–82.4)	70.6% (68.7–72.7)
G3b	81.9% (79.2–84.7)	73.9% (70.8–77.2)	61.4% (57.8–65.2)
G4	74.8% (68.3–82.0)	62.8% (55.5–71.1)	45.7% (38.0– 54.8)
G5	77.2% (73.0–81.7)	61.7% (56.8–67.0)	51.9% (46.8–57.5)

Table S2. Kaplan-Meier estimates of 1-year, 3-year, and 5-year survival (% with 95% confidence intervals) stratified by preoperative chronic kidney disease (CKD) stage.

CKD stage	≤70 years	>70 years
G1-2	90.5% (90.0–91.0)	79.2% (78.3–80.1)
G3a	77.4% (74.1–80.9)	68.0% (65.7–70.5)
G3b	69.1% (62.9–75.8)	58.4% (54.2–63.0)
G4	58.9% (47.1–73.7)	37.6% (28.6–49.5)
G5	54.7% (48.9–61.1)	43.2% (33.6–55.4)

Table S3. Kaplan-Meier estimates of 5-year survival (% with 95% confidence intervals) stratified by chronic kidney disease (CKD) stage and preoperative age.

CKD stage	AKI status	1-year survival	3-year survival	5-year survival
G1–2	No AKI	98.0% (97.8–98.2)	95.0% (94.7–95.4)	90.8% (90.4–91.3)
G1–2	AKI	90.3% (89.6–91.0)	83.9% (83.0–84.8)	75.5% (74.5–76.6)
G3a	No AKI	94.6% (93.3–95.9)	88.0% (86.0–89.9)	79.6% (77.1–82.1)
G3a	AKI	84.0% (81.9–86.2)	74.7% (72.1–77.4)	62.6% (59.7–65.7)
G3b	No AKI	89.3% (85.7–93.1)	82.2% (77.7–87.0)	68.1% (62.5–74.2)
G3b	AKI	78.1% (74.5–82.0)	69.6% (65.4–73.9)	57.9% (53.4–62.8)
G4	No AKI	80.5% (69.2–93.6)	72.5% (59.9–87.8)	63.9% (50.3–81.2)
G4	AKI	75.4% (67.8–83.9)	61.6% (52.9–71.6)	40.4% (31.6–51.5)
G5	No AKI	78.8% (68.4–90.8)	68.4% (56.6–82.6)	56.1% (43.4–72.5)
G5	AKI	78.2% (72.9–83.9)	59.7% (53.4–66.8)	52.0% (45.5–59.5)

Table S4. Kaplan-Meier survival estimates stratified by baseline chronic kidney disease (CKD) stage and postoperative acute kidney injury (AKI) status. Survival probabilities at 1, 3, and 5 years after cardiac surgery are presented as percentages with 95% confidence intervals in parentheses. Postoperative AKI was defined according to KDIGO criteria as either a plasma creatinine increase of ≥26.5 μmol/L within 48 hours or a ≥1.5-fold increase from baseline within seven days of surgery. Analysis includes 27,214 patients with available AKI data out of 27,483 total cardiac surgery patients.

Outcome	AKI status	Timepoint	Event incidence	Death incidence
Rapid progression	No AKI	1 year	7.3% (6.0–8.7)	6.5% (5.2–7.8)
Rapid progression	No AKI	3 years	24.7% (22.4–26.9)	11.0% (9.4–12.7)
Rapid progression	No AKI	5 years	36.0% (33.4–38.6)	14.6% (12.7–16.5)
Rapid progression	AKI	1 year	11.7% (10.1–13.2)	17.6% (15.8–19.5)
Rapid progression	AKI	3 years	32.4% (30.1–34.7)	22.5% (20.5–24.5)
Rapid progression	AKI	5 years	41.7% (39.2–44.1)	26.4% (24.2–28.6)
CKD progression	No AKI	1 year	2.4% (1.6–3.3)	6.8% (5.5–8.1)
CKD progression	No AKI	3 years	10.1% (8.5–11.7)	12.3% (10.6–14.0)
CKD progression	No AKI	5 years	16.5% (14.5–18.5)	18.9% (16.7–21.0)
CKD progression	AKI	1 year	9.7% (8.3–11.1)	17.6% (15.8–19.4)
CKD progression	AKI	3 years	23.2% (21.1–25.3)	24.0% (22.0–26.1)
CKD progression	AKI	5 years	30.7% (28.4–33.0)	30.1% (27.8–32.4)
Kidney failure	No AKI	1 year	0.2% (0.0–0.5)	6.7% (5.4–8.0)
Kidney failure	No AKI	3 years	1.2% (0.6–1.8)	13.2% (11.4–15.0)
Kidney failure	No AKI	5 years	2.3% (1.5–3.1)	22.2% (19.9–24.4)
Kidney failure	AKI	1 year	2.8% (2.0–3.6)	17.7% (15.8–19.5)
Kidney failure	AKI	3 years	5.6% (4.5–6.7)	25.9% (23.7–28.0)
Kidney failure	AKI	5 years	8.4% (7.0–9.8)	35.9% (33.5–38.3)

Table S5. Cumulative incidence estimates for kidney outcomes (rapid progression, CKD stage progression, kidney failure) and death as a competing event, stratified by acute kidney injury (AKI) status, are presented as percentages with 95% confidence intervals in parentheses at 1, 3, and 5 years after cardiac surgery. Estimates were calculated using the Aalen-Johansen estimator to appropriately account for death as a competing risk. Analysis restricted to patients with baseline CKD stages G3a to G4 who had available AKI data and sufficient follow-up creatinine measurements to assess progression outcomes (n=3,144). Postoperative AKI was defined according to KDIGO criteria.

Outcome	AKI status	Timepoint	Event incidence	Death incidence
Rapid progression	CABG	1 year	9.9% (8.3–11.5)	9.9% (8.3–11.5)
		3 years	29.7% (27.3–32.2)	13.3% (11.5–15.1)
		5 years	40.2% (37.5–42.9)	17.0% (15.0–19.0)
	Valve	1 year	9.6% (7.7–11.6)	11.7% (9.6–13.8)
		3 years	29.2% (26.2–32.2)	17.7% (15.2–20.2)
		5 years	39.5% (36.1–42.8)	21.9% (19.2–24.7)
	Combined	1 year	8.7% (6.4–10.9)	19.4% (16.3–22.6)
		3 years	27.4% (23.8–31.0)	24.1% (20.7–27.5)
		5 years	38.1% (34.1–42.1)	27.8% (24.2–31.4)
CKD progression	CABG	1 year	6.2% (4.9–7.5)	10.0% (8.5–11.6)
		3 years	17.4% (15.4–19.5)	14.6% (12.7–16.5)
		5 years	25.6% (23.2–28.0)	20.9% (18.7–23.2)
	Valve	1 year	7.0% (5.3–8.7)	11.5% (9.4–13.5)
		3 years	18.3% (15.7–20.9)	18.5% (15.9–21.0)
		5 years	24.8% (21.8–27.7)	25.5% (22.5–28.4)
	Combined	1 year	5.8% (3.9–7.7)	19.8% (16.6–22.9)
		3 years	15.9% (12.9–18.9)	27.1% (23.6–30.7)
		5 years	22.2% (18.8–25.7)	32.9% (29.1–36.8)
Kidney failure	CABG	1 year	1.5% (0.8–2.1)	9.9% (8.3–11.5)
		3 years	3.7% (2.7–4.7)	16.1% (14.1–18.0)
		5 years	5.8% (4.5–7.0)	25.1% (22.7–27.5)
	Valve	1 year	1.9% (1.0–2.8)	11.7% (9.6–13.8)
		3 years	3.5% (2.3–4.7)	20.7% (18.0–23.4)
		5 years	5.2% (3.7–6.7)	31.5% (28.3–34.7)
	Combined	1 year	1.0% (0.2–1.8)	19.9% (16.8–23.1)
		3 years	2.6% (1.3–3.8)	27.5% (23.9–31.1)
		5 years	4.9% (3.1–6.7)	37.1% (33.2–41.1)

Table S6. Cumulative incidence estimates for kidney outcomes (rapid progression, CKD stage progression, kidney failure) and death as a competing event, stratified by surgery type, presented as percentages with 95% confidence intervals at 1, 3, and 5 years after cardiac surgery. Estimates were calculated using the Aalen-Johansen estimator to appropriately account for death as a competing risk. Analysis restricted to patients with baseline CKD stages G3a to G4 who had sufficient follow-up creatinine measurements to assess progression outcomes. CABG, isolated coronary artery bypass grafting (n=1,382); Valve, isolated aortic or mitral valve replacement/repair (n=906; 646 aortic, 260 mitral); Combined, concurrent CABG and valve surgery or other combination procedures (n=615).
